# The missing evidence: Stricturing Crohn’s disease in clinical trials, a systematic review

**DOI:** 10.1093/ibd/izag079

**Published:** 2026-05-14

**Authors:** Elettra Merola, Daniele La Rocca, Mariano Scaglione, Giovanni Mario Pes, Maria Pina Dore

**Affiliations:** Department of Medicine, Surgery and Pharmacy, University of Sassari, Sassari 07100, Italy; Department of Medicine, Surgery and Pharmacy, University of Sassari, Sassari 07100, Italy; Department of Medicine, Surgery and Pharmacy, University of Sassari, Sassari 07100, Italy; Department of Radiology, James Cook University Hospital, Teesside University, Middlesbrough TS4 3BW, United Kingdom; Department of Medicine, Surgery and Pharmacy, University of Sassari, Sassari 07100, Italy; Department of Medicine, Surgery and Pharmacy, University of Sassari, Sassari 07100, Italy; Department of Medicine, Baylor College of Medicine, Houston, TX 77030, United States

**Keywords:** stricturing Crohn’s disease, biologic, small molecule, systematic review

## Abstract

**Background:**

Stricturing Crohn’s disease (CD) represents a major cause of morbidity, with limited prospective data addressing response to treatment. We performed a systematic review to evaluate the efficacy of biologic and small-molecule therapies in stricturing CD and to assess the role of imaging modalities in diagnosing and monitoring bowel stenoses.

**Methods:**

We conducted a systematic search of PubMed, Scopus, and Web of Science through August 26, 2025. Prospective studies, including randomized controlled trials and prospective observational cohorts, reporting outcomes in adult patients with stricturing CD treated with biologics or small molecules were included. Risk of bias was assessed using the RoB 2 and ROBINS-I tools.

**Results:**

Of 22 306 records identified, 7 publications corresponding to 10 prospective studies (6 randomized controlled trials, 4 nonrandomized studies) were included. All studies evaluated biologic therapies, predominantly anti-tumor necrosis factor agents; no prospective trials specifically assessed small-molecule therapies in stricturing CD. Due to methodological heterogeneity, a meta-analysis was not appropriate. Across studies, biologic therapy was associated with clinical improvement and, in selected cohorts, reductions in inflammatory activity and need for surgery. Response appeared to vary according to stricture severity, with lower rates in nonpassable lesions. Intestinal ultrasonography and magnetic resonance were investigated in 4 studies, showing reductions in bowel wall thickness.

**Conclusions:**

Prospective evidence for medical therapy in stricturing CD is limited and restricted to anti-tumor necrosis factor α agents. Imaging plays an important role in characterization and monitoring but lacks a standardized fibrosis assessment. Dedicated phenotype-specific trials and validated imaging biomarkers are needed to optimize management of stricturing CD.

## Graphical Abstract

**Graphical Abstract:**
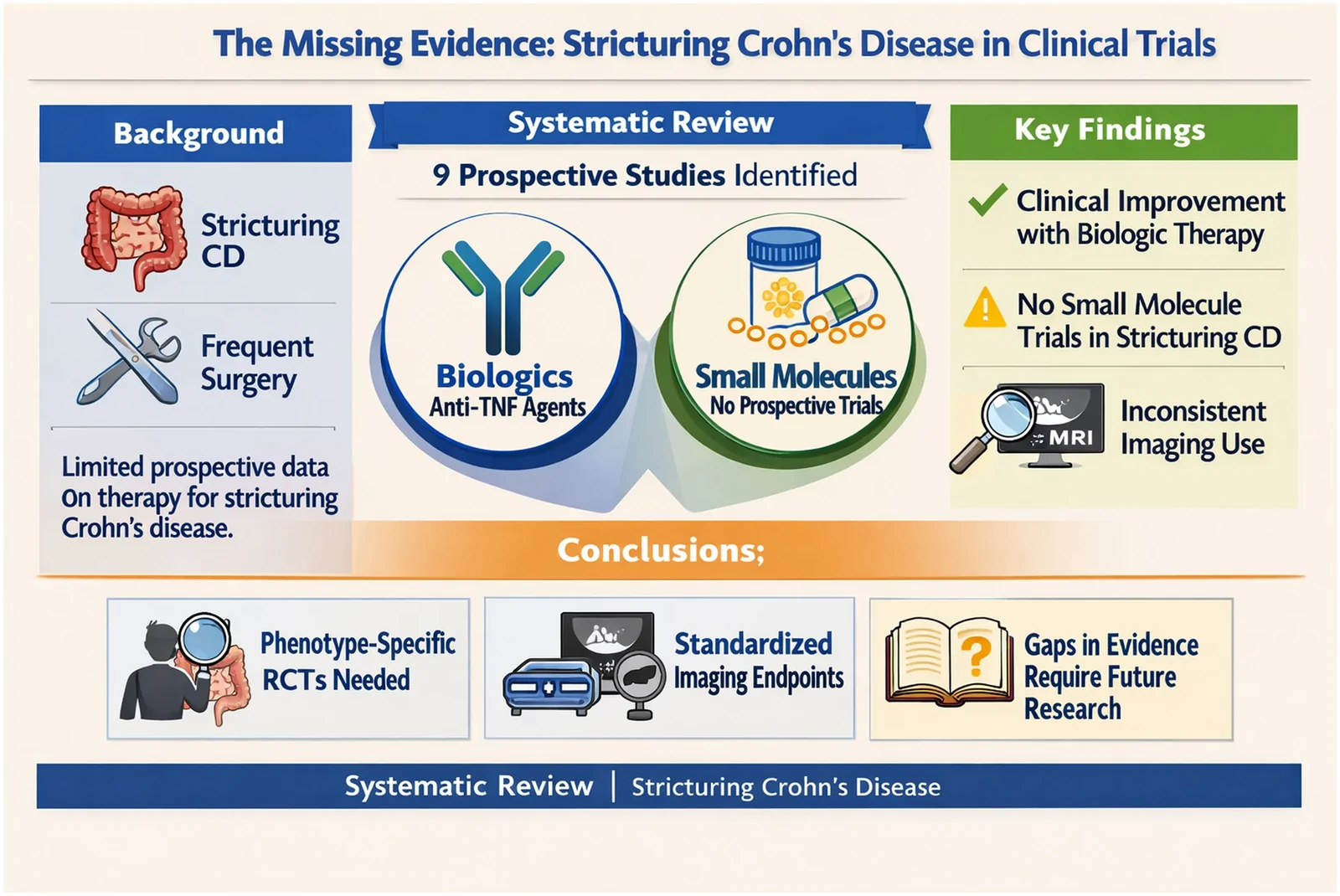

Key Messages **What is already known?**Most clinical trials enroll mixed populations without phenotype-specific analyses, limiting evidence for stricturing disease management.

**What is new here?**Only 7 prospective studies focused on this topic, while no prospective small-molecule trials were identified.

**How can this study help patient care?**Biologic therapy, particularly anti-tumor necrosis factor agents, may benefit selected patients with stricturing Crohn’s disease, but careful phenotypic assessment and structured imaging follow-up are essential.

## Introduction

Crohn’s disease (CD) is a chronic, immune-mediated inflammatory bowel disease (IBD) that may affect any part of the gastrointestinal tract, most commonly the terminal ileum. Its presentation and prognosis are highly variable, and disease behavior can change over time. In the Montreal classification, CD is described as inflammatory (nonstricturing, nonpenetrating), stricturing, or penetrating.^1^ Among these phenotypes, strictures involve almost 30% of patients at diagnosis and are a major source of morbidity, frequently causing obstructive symptoms, reducing quality of life, and often needing endoscopic or surgical management.^2–4^

In the prebiologic era, when treatment largely relied on corticosteroids and conventional immunomodulators, surgery was often the only effective option for symptomatic narrowing.^5^ However, recurrence after resection was frequent, making long-term monitoring and repeat interventions common.^6^ The introduction of anti-tumor necrosis factor (TNF) antibodies substantially changed the therapeutic landscape of CD patients. Initially, concerns that biologic therapy might worsen obstruction or accelerate fibrosis in already narrowed segments limited their use in stricturing disease, but subsequent data disproved this theory.^7–9^ Since then, significant advances in the medical management of CD have occurred, with the inclusion of additional biologic agents (such as anti-integrin agents and interleukin-12/23 inhibitors) as well as oral small molecules (including Janus kinase inhibitors and sphingosine-1-phosphate receptor modulators).^10^ However, stricturing CD remains difficult to manage: drug therapy may help selected patients and, in some cases, postpone surgery, but many individuals still require endoscopic balloon dilation or resection, and the best medical approach for established luminal narrowing remains uncertain.^11^

A major challenge lies in distinguishing the relative contribution of active inflammation and established fibrosis within a stricture, as this balance is likely to influence responsiveness to medical therapy.

Although endoscopy, intestinal ultrasonography (IUS), computed tomography enterography (CTE), and magnetic resonance enterography (MRE) are routinely used in practice, there is still no widely accepted in vivo reference standard or fully validated scoring system to quantify intestinal fibrosis. Differences in definitions and assessment methods across studies further complicate both clinical decision making and comparison of research findings.^12^

Within this context, this systematic review summarizes prospective evidence, including randomized controlled trials and prospective observational studies, reporting outcomes in patients with stricturing CD treated with biologic agents or small-molecule therapies. As a secondary objective, the study assesses the application of imaging modalities within the included prospective studies for detecting and monitoring bowel stenoses.

## Methods

The primary endpoint of this systematic review was to determine treatment response (clinical/or endoscopic, and/or biochemical activity) in patients with stricturing CD treated with biologic agents or small-molecule therapies in prospective studies. The secondary endpoint was to compare diagnostic imaging techniques (eg, IUS, CTE, and MRE) for (1) identifying CD-related strictures and/or (2) monitoring stricture-level response in the included prospective studies.

We conducted a systematic search of PubMed, Scopus, and Web of Science, last updated August 26, 2025. For “patient selection,” the following words were adopted in the query: (((“Inflammatory Bowel Diseases”[Mesh] OR “Crohn Disease”[Mesh] OR “Colitis, Ulcerative”[Mesh] OR IBD OR “inflammatory bowel disease” OR “crohn’s disease” OR “ulcerative colitis”) AND (strictur* OR stenosis OR substenosis OR fibrotic OR fibrosis OR fibrostenos* OR “fibro-stenotic” OR narrowing OR obstruction OR “intestinal obstruction”))).

For “therapy type,” these terms were used: (“Biological Therapy”[Mesh] OR biologic* OR “anti-TNF” OR infliximab OR adalimumab OR vedolizumab OR ustekinumab OR risankizumab OR upadacitinib OR tofacitinib OR ozanimod OR etrasimod OR “small molecule*” OR “JAK inhibitor*” OR “S1P modulator*”).

Complete search queries are reported in Supplemental Material 1.

Handsearching using conference papers and bibliography of included studies was also performed.

Inclusion and exclusion criteria, as well as PICO (patient, intervention, comparison, outcome) strategy, are summarized in Supplemental Material 2. Regarding disease selection, exclusion criteria were ulcerative colitis, nonstricturing CD, stricturing CD treated with other therapies than biologics or small molecules, and age <18 years.

Studies enrolling mixed IBD populations were excluded unless CD-specific results were reported separately. We also excluded studies that included patients with CD but did not provide outcomes stratified by disease phenotype.

Regarding study type, the following were excluded: narrative or systematic reviews; case reports and series <10 patients; retrospective studies; preclinical or laboratory investigations without human participants correspondence, editorials, guidelines, and position papers.

Two reviewers (E.M., D.L.R.) independently screened titles/abstracts in Rayyan (Rayyan Systems Inc.).^13^ Disagreements were resolved by a third reviewer (M.P.D.). Full texts of potentially eligible records were then assessed against the inclusion criteria. Reasons for exclusion at the full-text stage were recorded. We used the published data without contacting the study authors.

Study selection was performed according to the PRISMA (Preferred Reporting Items for Systematic Reviews and Meta-Analyses) 2020 guidelines,^14^ and once eligible studies were selected, the data extraction for quantitative analysis was completed by 2 authors (E.M., D.L.R.), using the data reported in the publications.

For each included study, we extracted first author, publication type and year, total sample size, number of participants with stricturing disease, treatment details (agent, dose, schedule), outcome definitions and results, and the imaging/diagnostic modality used at baseline and/or follow-up to characterize strictures.

Data about the outcomes were recorded, when possible, as dichotomous variables (presence or absence of the outcome).

Two reviewers (E.M., D.L.R.) independently appraised study quality. We adopted the RoB 2 for randomized trials and ROBINS-I for nonrandomized prospective studies.^15^^,^^16^ Discrepancies were adjudicated by a third reviewer (M.P.D.).

The review protocol was registered in the PROSPERO database (CRD420251139457).

Language editing of the manuscript was performed using an artificial intelligence–based language model (ChatGPT, OpenAI), as well as preparation of graphical abstract.

## Results

### Study selection and risk-of-bias assessment

A total of 22 306 records were identified (Figure 1). After removing 7932 duplicates, 14 374 titles and abstracts were screened. Of these, 12 463 records were excluded and 1911 full-text articles were assessed for eligibility. Ultimately, 7 records were eligible for the final analysis.^17–23^ They corresponded to 10 prospective studies, comprising 6 randomized^18^^,^^19^^,^^24–26^ and 4 nonrandomized studies,^17^^,^^20^^,^^22^^,^^23^ evaluating treatment response in patients with stricturing CD.

**Figure 1:**
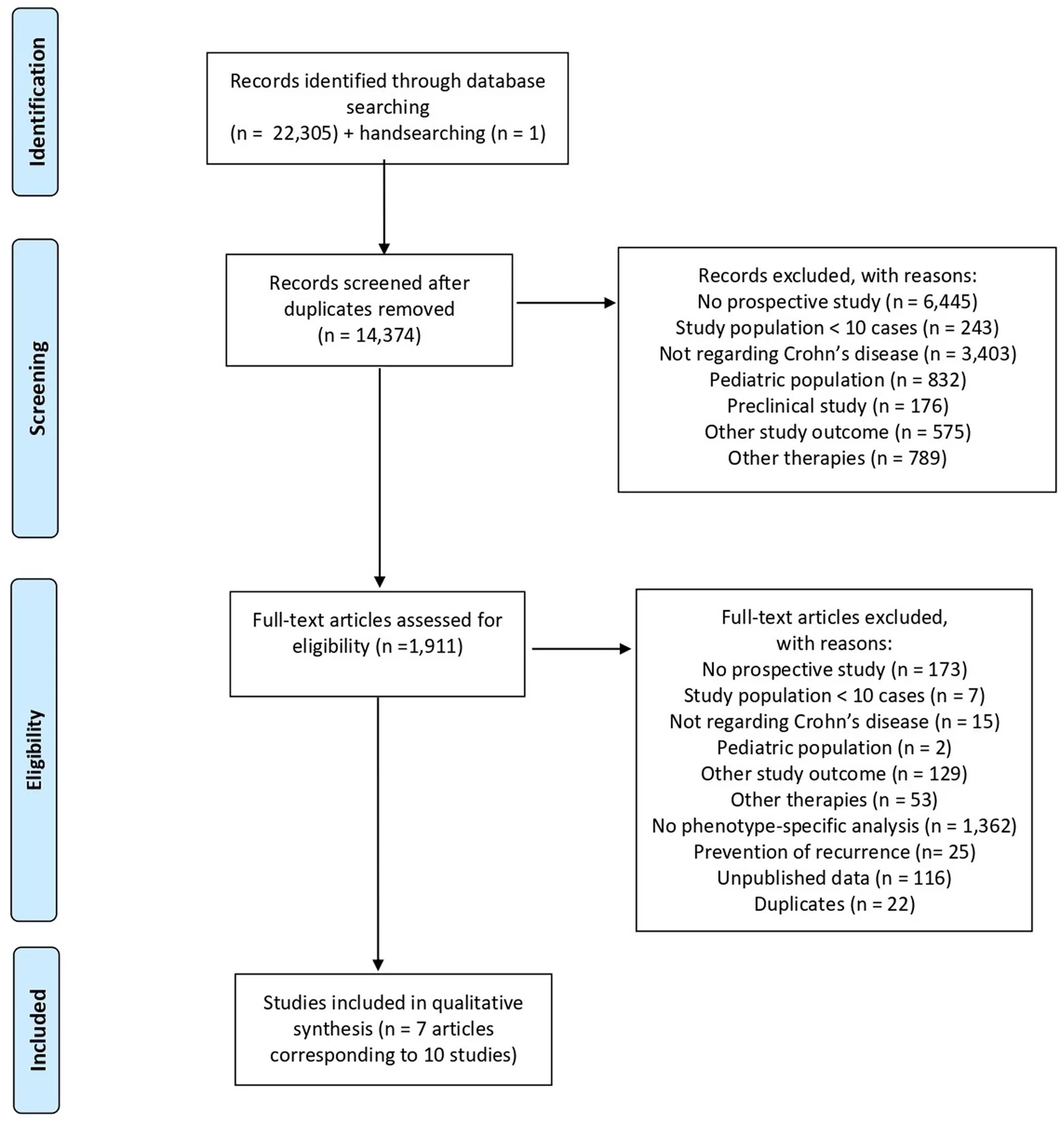
PRISMA (Preferred Reporting Items for Systematic Reviews and Meta-Analyses) flow chart for study selection.

The overall risk-of-bias profile clearly distinguishes between randomized and nonrandomized evidence (Figure 2). Among the randomized controlled trials, methodological quality was generally acceptable. Two trials^25^^,^^26^ were judged at an overall low risk of bias across domains, reflecting robust randomization procedures, adequate blinding, minimal missing data, and consistency between prespecified and reported outcomes. In contrast, the SONIC (Study of Biologic and Immunomodulator Naive Patients in Crohn's Disease)^24^ and the STRIDENT (Intensive drug therapy versus standard drug therapy for symptomatic intestinal Crohn's disease strictures) studies^18^^,^^19^ were rated as having some concerns overall, primarily driven by potential deviations from intended interventions and, in the case of the SONIC study, missing outcome data. Nevertheless, no high-risk judgments were assigned within the RoB 2 assessment, supporting the relative internal validity of the randomized evidence.

**Figure 2:**
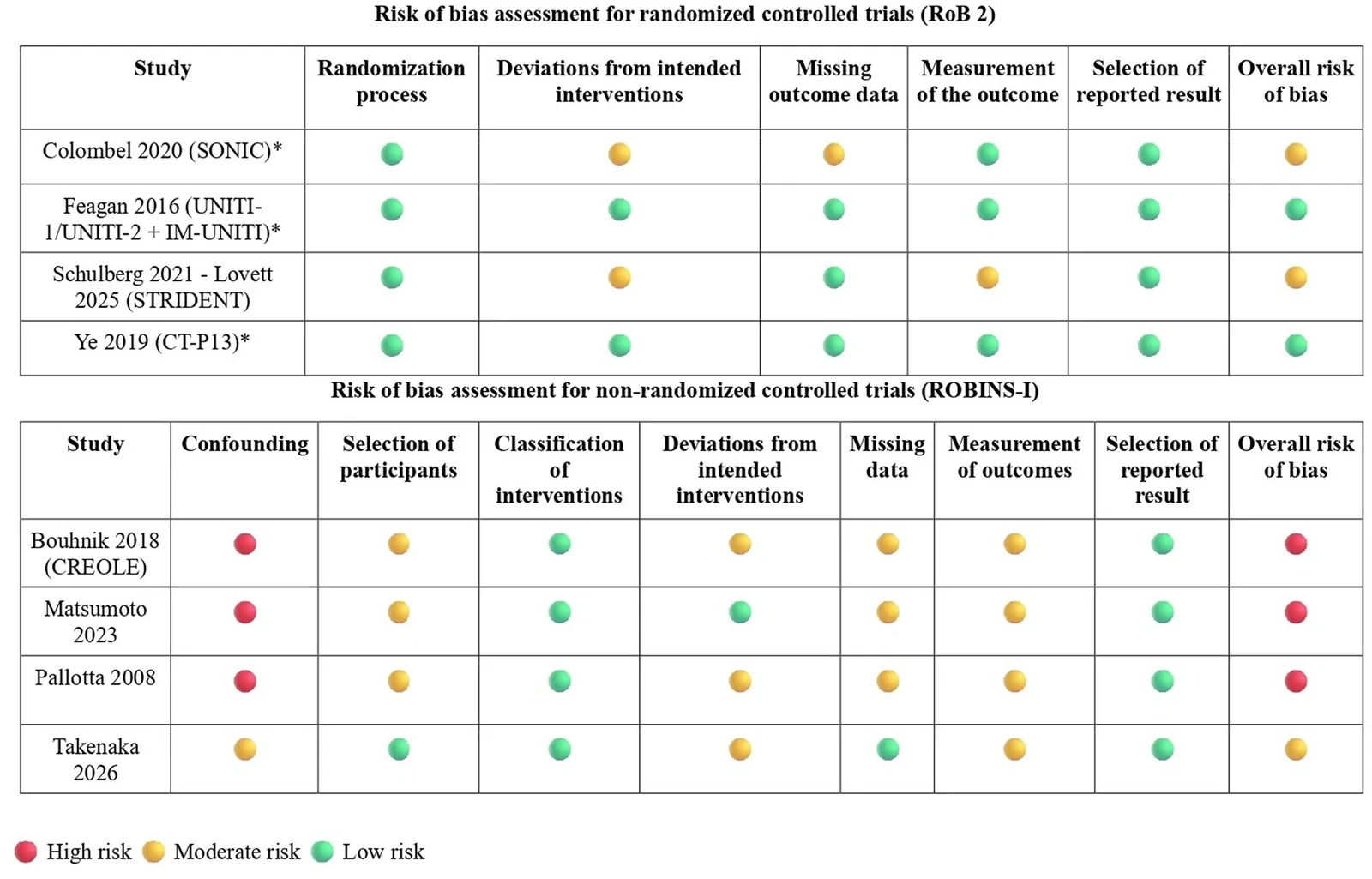
Risk-of-bias assessment for randomized controlled trials (RoB 2) and nonrandomized trials (ROBINS-I). Data from trials marked with an asterisk were derived from a post hoc analysis published by Narula et al.^21^ The red circle indicates a high risk of bias, the green circle a low risk of bias, and the yellow circle a moderate risk of bias.

By contrast, most nonrandomized studies assessed with ROBINS-I were judged at overall high risk of bias.^17^^,^^22^^,^^23^ In each case, a serious confounding risk was identified, reflecting the inherent limitations of observational designs in the absence of random allocation. Although classification of interventions and selective reporting were generally considered low risk, moderate concerns persisted in domains such as participant selection, deviations from intended interventions, missing data, and outcome measurement.

Given these considerations and the substantial heterogeneity across the included studies, particularly in the therapeutic regimens evaluated and the outcome definitions, a quantitative meta-analysis was not appropriate. We therefore performed a descriptive synthesis to outline overall patterns of treatment response within the different drug classes and dosing strategies.

### Primary endpoint: Response to therapy

All included studies investigated biologic therapies, predominantly anti-TNFα agents, and consistently reported clinical improvement in patients with stricturing CD (Tables 1 and 2).^17–23^ No prospective trials specifically designed to assess small-molecule therapies in stricturing CD were identified.

**Table 1:** Study characteristics and endpoints of the included prospective studies.

Study	Study design	Population	Study drug	Prohibited concomitant treatments	Primary endpoints	Secondary endpoints	Reference standard for disease activity/severity
**Bouhnik 2018** ^17^ **: CREOLE**	Prospective, multicenter, open-label, observational, cohort study	CD with symptomatic small bowel stricture (but excluding surgical obstruction)	Adalimumab 160 mg at baseline → 80 mg at week 2 → 40 mg every other week	- Steroids after 8 wk following inclusion - Other anti-TNF - Endoscopic dilation - Bowel resection	- Success at week 24 (no need of prohibited drugs or other intervention, no toxicity) - Definition of a prognostic score	- Success after week 24 - Time to bowel resection in the whole cohort	- CDAI - CDOS - CRP
**Matsumoto 2023** ^23^	Prospective pilot study	Patients with stricturing CD	- Infliximab - Ustekinumab - Infliximab → ustekinumab	Not defined	To evaluate the change in bowel thickness and stiffness after treatment	Clinical and biochemical response	- CDAI - CRP
**Narula 2021** ^21^	Prospective post hoc analysis of 5 trials (SONIC + UNITI-1 + UNITI-2 + IM-UNITI + CT-P13)	CD with nonpassable and passable strictures	- Infliximab 5 mg/kg at regular time vs azathioprine 2.5 mg/kg vs infliximab 5 mg/kg + azathioprine 2.5 mg/kg - Ustekinumab 130 mg or ustekinumab 6 mg/kg vs placebo → ustekinumab subcutaneous 90 mg (either every 8 or 12 wk) vs placebo - CT-P13 → CT-P13 at week 30 vs CT-P13 → infliximab at week 30 vs infliximab → infliximab at week 30 vs infliximab → CT-P13 at week 30	Immediate need of surgery and balloon dilation	Conversion of nonpassable stenoses in passable or no stenoses after 1 y of medical treatment	- Clinical remission/response - Endoscopic remission/response	- CDAI - SES-CD - Endoscopic evaluation
**Pallotta 2008** ^22^	Long-term, single-center, prospective follow-up study	CD receiving infliximab	Infliximab 5 mg/kg intravenous at weeks 0, 2, and 6, then 5 mg/kg every 8 wk	—	To morphologically assess the therapeutic response of small bowel strictures	- Change in obstructive symptoms - Need for surgery during infliximab - Changes in CDAI - Occurrence of new stenoses during infliximab	- CDAI - IUS
**Schulberg 2022,^18^ Lovett 2025^19^: STRIDENT**	Open-label, single-center, randomized controlled trial	CD patients with symptomatic small bowel strictures (with active inflammation)	- Standard arm: adalimumab 160 mg at baseline → 80 mg at week 2 → 40 mg every 2 wk - Intensive arm: adalimumab (160 mg weekly for 4 wk → 40 mg every 2 wk) + azathioprine (2.5 mg/kg) or mercaptopurine (1.5 mg/kg) daily with dose adjustment	- Urgent surgical resection (while endoscopic dilation before start allowed) - Steroids after 4 wk from baseline - Nonstudy thiopurines. Tacrolimus, thalidomide, cyclosporine, other biologics, NSAIDs	↓ symptoms at 12 mo	- Clinical, laboratory, radiological improvement - Safety in stricture inflammation and morphology on imaging	- Endoscopy - CDAI - CDOS - IBDQ - CRP - Fecal calprotectin - U.S. (Doppler) MRE
**Takenaka 2026** ^20^	Post hoc analysis of prospective, real-world study	CD patients with stricturing disease	Anti-TNF, ustekinumab or vedolizumab	—	Endoscopic change of strictures	- Clinical remission - Biochemical remission - Prognostic factors	- Endoscopy - SES-CD - CDAI - CRP - Fecal calprotectin

Abbreviations: CD, Crohn’s disease; CDAI, Crohn’s disease activity index; CDOS, Crohn’s disease obstructive score; CREOLE, •••; CRP, C-reactive protein; IBDQ, inflammatory bowel disease questionnaire; IUS, intestinal ultrasonography; MRE, magnetic resonance enterography; NSAID, nonsteroidal anti-inflammatory drug; SES-CD, Simple Endoscopic Score for Crohn’s Disease; SONIC, •••; STRIDENT, •••; TNF, tumor necrosis factor; UNITI, •••.

**Table 2:** Efficacy and safety outcomes of biologic therapy in stricturing CD across included studies.

Study	B2^a^	Stricture location^a^	Follow-up	Primary outcomes	Secondary outcomes	Adverse events
**Bouhnik 2018** ^17^ **: CREOLE**	97 (100)	- L1: 69 (71.1) - L3: 19 (19.6) - L4: 9 (9.3)	Median 3.8 ± 0.1 y	- Success at week 24: 62 (63.9%) - Prognostic variables: immunosuppressive drugs at adalimumab start, obstructive symptoms for >5 wk, CDOS >4; at MRE: stricture <12 cm, max diameter proximal to stricture 18-29 mm, marked enhancement on delayed phase, no fistulae	- Success at 4 y: 45.7 ± 6.6% - 4-y surgery-free survival; 50.7 ± 5.3%	- SAE: 72.2% - Surgery at week 24: 8.2% - Endoscopic dilation at week 24: 2.1% - Surgery after 24 wk: 74.2%
**Matsumoto 2023** ^23^	21 (100)	- L1: 2 (9.5) - L3: 19 (90.5)	12 mo	- Median change in bowel wall thickness: −1.51 with infliximab (*P* = .02) vs −1.03 with ustekinumab (*P* = .01) vs −1.2 in the bio-switch group (*P* = .02) - Median change in shear wave speed: −0.22 with infliximab (*P* = .71) vs −1.58 with ustekinumab (*P* = .03) vs −0.28 in the bio-switch group (*P* = .92)	- ↓ CDAI and CRP after infliximab -↓ CRP after ustekinumab - ↓ CDAI in the bio-switch group - Duration of biologics treatment longer and operation history more frequent in the bio-switch group	—
**Narula 2021** ^21^	150 (26.0)	- L1: 82 (54.7) - L2: 68 (45.3)	12 mo	From nonpassable stenosis to passable/no stenosis at 1 y: 62.5%	− Clinical response: 57.1% for nonpassable stenosis vs 69.9% for passable/no stenosis (*P* = .01)	—
**Pallotta 2008** ^22^	10 (66.7)	—	34.7 ± 16.1 mo	- Complete stricture resolution: 60.0%	- Need for surgery during infliximab mantainance therapy 2 (20.0) - ↓ CDAI: 90.0% - Occurrence of new stenoses during infliximab treatment: 0%	—
**Schulberg 2022,^18^ Lovett 2025^19^**	77 (100): 25 in standard arm vs 52 in intensive arm	- L1: 52 (67.5) - L2: 3 (3.9) - L3: 21 (27.3) - L4: 1 (1.3)	12 mo	− Overall clinical improvement: 74.0%	- ↓ CDAI: 66.2% - ↓ CDOS: 57.1% - ↓ CRP: 59.7% - ↓ Fecal calprotectin: 64.9% reduction - Benefit from intensive treatment only on imaging	- Total vent (overall): 88.3% - SAE: 15.6% - Surgical intervention: 10.4% (4 cases per arm)
**Takenaka 2026** ^20^	170 (100)	- L1: 89 (52.4) - L3: 81 (46.4)	40 mo (IQR: 32–47 mo)	Resolution rates: 34.4% for anti-TNF, 23.5% for ustekinumab, 40.0% for vedolizumab - Changes in the endoscopic findings according to drug: *P* = .44	- Clinical remission: 91.8% - Biochemical remission: 55.9% - SES-CD: 11 → 4	- Bowel obstruction: 7.0% - Intestinal bleeding: 0.6% - Surgery: 7.0%

Values are n (%) or mean ± SD, unless otherwise indicated.

Abbreviations: CD, Crohn’s disease; CDAI, Crohn’s disease activity index; CDOS, Crohn’s disease obstructive score; CRP, C-reactive protein; IQR, interquartile range; MRE, magnetic resonance enterography; SAE, serious adverse event; SES-CD, Simple Endoscopic Score for Crohn’s Disease; TNF, tumor necrosis factor.

aAccording to Montreal classification.

The CREOLE prospective cohort enrolled 97 patients with symptomatic small bowel strictures treated with adalimumab.^17^ At week 24, the predefined primary endpoint of “success” was achieved in 63.9% of patients. Success was defined as continuation of adalimumab without the need for prohibited medications, endoscopic dilation, bowel resection, treatment discontinuation due to severe adverse events, or withdrawal from the study. Among responders, 88% achieved a Crohn’s Disease Activity Index <150, indicating meaningful clinical remission. Importantly, long-term follow-up showed that nearly half of the cohort remained free from surgery 4 years after treatment initiation.

Similarly, in the STRIDENT randomized trial,^18^^,^^19^ patients with symptomatic stricturing CD were allocated (2:1) to intensive high-dose adalimumab plus thiopurine or to standard-dose adalimumab monotherapy. At 12 months, 74% of the overall cohort experienced improvement in obstructive symptoms, accompanied by reductions in inflammatory biomarkers and clinical disease activity. Although outcomes numerically favored the intensive regimen, between-group differences did not reach statistical significance, except for a lower rate of treatment failure in the intensive arm (*P* = .04).

Takenaka et al^20^ evaluated the evolution of ileal strictures during biologic therapy in a post hoc analysis of a prospective real-world cohort including 170 patients with ileal or ileocolonic CD initiating or switching biologic treatment (anti-TNF agents, ustekinumab, or vedolizumab). Strictures were assessed endoscopically using balloon-assisted enteroscopy at baseline and after 6 months. At follow-up, stricture resolution and progression were observed in 30.9% and 15.7% of cases, respectively. When stratified according to the biologic agent used, resolution occurred in 34.4% (n = 11 of 32) of patients treated with anti-TNF agents, 23.5% (n = 8 of 34) of those receiving ustekinumab, and 40.0% (n = 6 of 15) of patients treated with vedolizumab, whereas progression was reported in 14.3% (n = 6 of 42), 16.0% (n = 4 of 25), and 18.2% (n = 4 of 22), respectively. However, no statistically significant differences were observed among biologic classes with regard to either stricture resolution (*P* = .443) or progression (*P* = .167). Importantly, while the presence of strictures at baseline was not associated with clinical outcomes, the persistence of strictures at 6 months—particularly in the nonterminal ileum—was linked to a higher risk of hospitalization and surgery during follow-up.

Finally, Narula et al^21^ conducted a post hoc analysis of 5 randomized trials from 3 major CD clinical trial programs involving infliximab and ustekinumab,^24–26^ focusing on patients with endoscopically defined strictures. Among 576 participants, 105 had nonpassable and 45 had passable strictures at baseline. After 1 year of therapy, 62.5% of patients with nonpassable strictures improved to a passable or no stricture status. Clinical remission was achieved in 52.4% of these patients, and endoscopic remission in 37.5%. However, outcomes were not stratified according to the specific biologic agent, limiting treatment-specific conclusions.

### Secondary endpoint: Role of imaging

In prospective studies enrolling patients with stricturing CD, radiologic assessment has progressively evolved from a simple eligibility tool to an integral component for monitoring stricture-level response during medical therapy (Table 3).

**Table 3:** Secondary outcome: Imaging modalities and their use in included studies.

Study	Imaging	Timing	Role of imaging in the study	Outcomes
**Bouhnik 2018** ^17^ **: CREOLE**	MRE	Baseline	- Eligibility confirmation - Collection of baseline imaging features for clinic-radiological prognostic score - No imaging-based response endpoint	—
**Matsumoto 2023** ^23^	IUS with elastography	- Baseline - After 1 y	- Stricture definition - Response assessment	- To assess the change in bowel wall thickness and stiffness of strictures
**Pallotta 2008** ^22^	IUS	- Baseline - Every 6 mo	- Stricture definition - Response assessment	To assess the change in bowel wall thickness
**Schulberg 2022,^18^ Lovett 2025^19^: STRIDENT**	- MRE - IUS with Doppler	- Baseline and at 12 mo - Baseline and after 8 and 12 mo	- Eligibility confirmation - Response assessment	- Stricture resolution on MRI: 22.2% - Stricture improvement on MRE at 12 mo: 33.3% - Stricture resolution on IUS: 28.8% - Stricture improvement on IUS at 12 mo: 39.0% - Correlation MRE and IUS: Cramer’s V ϕ = 0.84 (*P* < .001).
**Takenaka 2026** ^20^	Cross-sectional imaging (including MRE)	Baseline	Not required for the study definition of strictures	—

Abbreviations: CREOLE, •••; IUS, intestinal ultrasonography; MRE, magnetic resonance imaging; MRI, magnetic resonance imaging; STRIDENT, •••.

In a prospective single-center study, Pallotta et al^22^ evaluated the effects of infliximab on small bowel strictures in 10 patients with CD, using IUS as the primary imaging modality for longitudinal follow-up during both induction and maintenance therapy. Serial IUS examinations showed no progression of pre-existing strictures and no emergence of new stenoses. Notably, complete regression was documented in 8 (80.0%) of 10 strictures among patients who continued maintenance therapy, including lesions associated with prestenotic dilatation. In the remaining cases, strictures remained morphologically stable, and clinical symptoms improved despite the absence of radiologic progression. Although limited by its small sample size and observational design, this study provides early, prospective imaging-based evidence that anti-TNFα therapy may stabilize, and in selected cases, reverse, small bowel strictures, challenging earlier concerns about potential fibrotic worsening.

More recently, Matsumoto et al^23^ conducted a prospective pilot study assessing changes in stricture stiffness using ultrasound shear wave elastography in 21 patients treated with biologics. Patients were stratified into 3 groups: infliximab-naive, ustekinumab-naive, and those switching from infliximab to ustekinumab. After 12 months, bowel wall thickness decreased significantly across all groups, indicating reduced inflammatory activity. However, a significant reduction in shear wave speed, a surrogate marker of tissue stiffness, was observed only in the ustekinumab group (*P* = .03). This result may suggest that while biologic therapy may consistently reduce inflammatory wall thickening, its impact on structural stiffness, potentially reflecting fibrotic remodeling, could vary according to the therapeutic target. The small cohort size and absence of histological validation limit definitive conclusions.

In contrast, imaging in the CREOLE prospective cohort by Bouhnik et al^17^ was primarily used for baseline characterization. MRE was mandatory to confirm the presence of small bowel strictures and exclude contraindications to anti-TNFα therapy. However, imaging was not systematically incorporated as a longitudinal endpoint, no comparative evaluation of different modalities was performed, and MRE findings were recorded locally without central reading. Moreover, imaging results were not used to guide therapeutic decisions beyond confirming eligibility.

A more structured integration of imaging was implemented in the STRIDENT trial,^18^^,^^19^ in which active inflammatory strictures had to be documented by both MRE and IUS using predefined radiologic criteria. Imaging was embedded into follow-up: IUS was performed at baseline and every 4 months, while MRE was repeated at baseline and at 12 months. Although magnetic resonance imaging (MRI)–defined stricture resolution did not differ significantly between treatment arms or according to clinical response, IUS demonstrated that clinical responders achieved stricture resolution more frequently than nonresponders (37% vs 6%; *P* = .02). Resolution was also more common in patients with fecal calprotectin <100 μg/g at 12 months on both IUS and MRI (*P* < .01). Baseline IUS parameters predictive of 12-month resolution included bowel wall thickness <5 mm (odds ratio [OR], 5.3; *P* = .01) and stricture length <5 cm (OR: 3.9; *P* = .03). Additionally, a baseline MRI risk score of 0 was associated with a higher probability of stricture resolution (OR, 4.56; *P* = .02).

## Discussion

The present systematic review underscores that patients with stricturing CD remain an underrepresented and often overlooked subgroup within IBD research. Despite significant advances in the medical management of CD, our comprehensive search identified only 7 studies meeting predefined inclusion criteria.^17–23^ This scarcity likely reflects 2 major issues. First, most clinical trials enrolled heterogeneous CD populations without conducting phenotype-specific analyses. Second, patients with stricturing disease are often excluded from trials. As illustrated in Table S1, studies that explicitly include stricturing CD represent the exception rather than the norm.

One possible explanation is the long-standing perception that stricturing CD may respond poorly to medical therapy. This concern is supported by earlier evidence, such as the study by Hall et al,^27^ which found that stricturing disease was associated with limited endoscopic improvement and a high rate of subsequent surgical intervention. Discouraging results were also reported by a small open-label pilot study evaluating infliximab in patients with symptomatic, subocclusive small bowel strictures refractory to corticosteroids or immunosuppressive therapy. The trial was prematurely terminated after enrollment of 6 patients because predefined safety thresholds were reached, with more than 2 patients requiring early surgery. Only 2 participants completed the planned 8-week follow-up and showed clinical and biochemical improvement, whereas the remaining 4 ultimately required surgical intervention.^28^ Such findings may have contributed to the cautious approach traditionally adopted in clinical trials.

However, subsequent reports have challenged this assumption. A retrospective study evaluating infliximab in patients with CD reported encouraging outcomes in those with inflammatory strictures. ^29^ Among 21 treated patients, 11 presented with inflammatory stenoses, and the majority experienced marked clinical improvement with resolution of obstructive symptoms following anti-TNF therapy. These findings suggested that medical treatment may be effective in selected inflammatory strictures and could potentially delay or avoid surgical intervention, although the authors emphasized the need for prospective studies to confirm these observations.

The studies included in our analysis also reported encouraging outcomes.^17–23^ Across prospective analyses, biologic therapy, primarily anti-TNFα agents, was associated with meaningful clinical improvement in patients with stricturing CD. Only 1 study analyzed vedolizumab comparing it to anti-TNF and ustekinumab,^20^ but with discordant results: a higher resolution rate in comparison with anti-TNF and ustekinumab, and also a higher progression rate and no significant differences regarding endoscopic changes of strictures.

Nevertheless, not all strictures behave similarly. Post hoc data from Narula et al^21^ demonstrated that patients with nonpassable strictures had significantly lower response rates to biologics compared with those with passable or no strictures (57.1% vs 69.6%; *P* = .01), suggesting that stricture severity may influence therapeutic outcomes. An important aspect that warrants further emphasis is the complex histopathological nature of CD strictures. Intestinal stenoses are rarely purely fibrotic; rather, most lesions represent a dynamic interplay between chronic inflammation and extracellular matrix remodeling. The relative contribution of these components likely determines responsiveness to medical therapy, as predominantly inflammatory strictures may improve with anti-inflammatory treatment, whereas advanced fibrotic remodeling may be less reversible.

In this context, radiologic imaging becomes central to patient selection and monitoring. Techniques such as IUS, CTE, and MRE have shown comparable diagnostic accuracy for detecting strictures.^12^ Among the studies included in our review, only 4 incorporated imaging to support diagnosis and/or evaluate treatment response.^17–19^^,^^22^^,^^23^ Their results indicate that biologic therapy can induce changes not only in symptoms and inflammatory markers, but also in structural parameters such as bowel wall thickness and stiffness, and can occasionally lead to partial or complete stricture resolution. However, clinical trials do not stratify patients according to the inflammatory-fibrotic balance of the stricture. As a result, treatment decisions and trial interpretations are based on phenotypic labels that do not adequately reflect the underlying tissue composition, representing a major methodological gap in literature.

Notably, most available data concern IUS and MRE, while evidence regarding CTE remains limited, likely reflecting guideline recommendations favoring radiation-sparing techniques.^30^ Emerging technologies, including photon-counting CT, may expand future options by reducing radiation exposure and providing quantitative metrics, such as iodine density, that could serve as imaging biomarkers of disease activity.^31^ However, prospective validation studies are needed before these tools can be integrated into routine management.^32^

Regarding pharmacologic strategies, all the studies included in our systematic review focused on anti-TNF agents, particularly infliximab and adalimumab.^17–19^^,^^21–23^ These findings counter early concerns that anti-TNF therapy might exacerbate obstructive symptoms or precipitate complete occlusion in stricturing disease. At the same time, heterogeneity in dosing regimens, formulations, and outcome definitions precluded quantitative synthesis. While data regarding ustekinumab remain scanty, suggesting a potential benefit for this biological therapy,^21^^,^^23^ evidence for other biologic classes, including anti-integrin, anti-IL-23p19 agents, and small molecules in stricturing CD, is lacking, highlighting a substantial research gap.

In conclusion, current evidence on medical therapy for stricturing CD is limited and methodologically heterogeneous. Anti-TNFα agents appear to offer clinical benefit in selected patients, but careful phenotypic characterization is essential to optimize patient selection and minimize treatment failure. Radiologic imaging, particularly IUS and MRE, plays a critical role in both baseline assessment and longitudinal monitoring, but standardized, noninvasive biomarkers capable of distinguishing predominantly inflammatory from predominantly fibrotic strictures are still lacking.

Well-designed prospective trials evaluating newer biologics, small molecules, and imaging-guided treatment strategies are urgently needed to refine therapeutic algorithms and advance personalized management for patients with stricturing CD.

## Supplementary Material

izag079_Supplementary_Data

## References

[1] Silverberg MS , Satsangi J, Ahmad T, et al Toward an integrated clinical, molecular and serological classification of inflammatory bowel disease: report of a Working Party of the 2005 Montreal World Congress of Gastroenterology. Can J Gastroenterol. 2005;19(Suppl A):5A-36A. 10.1155/2005/269076 16151544

[2] Peyrin-Biroulet L , Loftus EV, Colombel JF, et al The natural history of adult Crohn’s disease in population-based cohorts. Am J Gastroenterol. 2010;105:289-297. 10.1038/ajg.2009.579 19861953

[3] Adamina M , Minozzi S, Warusavitarne J, et al ECCO guidelines on therapeutics in Crohn’s disease: surgical treatment. J Crohns Colitis. 2024;18:1556-1582. 10.1093/ecco-jcc/jjae089 38878002

[4] El Ouali S , Click B, Holubar SD, et al Natural history, diagnosis and treatment approach to fibrostenosing Crohn’s disease. United European Gastroenterol J. 2020;8:263-270. 10.1177/2050640620901960 PMC7184664 32213020

[5] Dehn TC , Kettlewell MG, Mortensen NJ, et al Ten-year experience of strictureplasty for obstructive Crohn’s disease. Br J Surg. 1989;76:339-341. 10.1002/bjs.1800760407 2720341

[6] Van Assche G , Dignass A, Reinisch W, et al; European Crohn’s and Colitis Organisation (ECCO). The second European evidence-based Consensus on the diagnosis and management of Crohn’s disease: special situations. J Crohns Colitis. 2010;4:63-101. 10.1016/j.crohns.2009.09.009 21122490

[7] Fiorino G , Bonifacio C, Allocca M, et al Bowel damage as assessed by the Lémann index is reversible on anti-TNF therapy for Crohn’s disease. J Crohns Colitis. 2015;9:633-639. 10.1093/ecco-jcc/jjv080 25958059

[8] D’Haens G , Van Deventer S, Van Hogezand R, et al Endoscopic and histological healing with infliximab anti-tumor necrosis factor antibodies in Crohn’s disease: a European multicenter trial. Gastroenterology. 1999;116:1029-1034. 10.1016/s0016-5085(99)70005-3 10220494

[9] Pelletier AL , Kalisazan B, Wienckiewicz J, et al Infliximab treatment for symptomatic Crohn’s disease strictures. Aliment Pharmacol Ther. 2009;29:279-285. 10.1111/j.1365-2036.2008.03887.x 19035967

[10] Gordon H , Minozzi S, Kopylov U, et al ECCO guidelines on therapeutics in Crohn’s disease: medical treatment. J Crohns Colitis. 2024;18:1531-1555. 10.1093/ecco-jcc/jjae091 38877997

[11] Zorzi F , Zuzzi S, Onali S, et al Efficacy and safety of infliximab and adalimumab in Crohn’s disease: a single centre study. Aliment Pharmacol Ther. 2012;35:1397-1407. 10.1111/j.1365-2036.2012.05100.x 22519466

[12] Dal Buono A , Faita F, Armuzzi A, et al Assessment of activity and severity of inflammatory bowel disease in cross-sectional imaging techniques: a systematic review. J Crohns Colitis. 2025;19:jjaf023. 10.1093/ecco-jcc/jjaf023 39901740

[13] Ouzzani M , Hammady H, Fedorowicz Z, et al Rayyan-a web and mobile app for systematic reviews. Syst Rev. 2016;5:210. 10.1186/s13643-016-0384-4 PMC5139140 27919275

[14] Page MJ , McKenzie JE, Bossuyt PM, et al [The PRISMA 2020 statement: an updated guideline for reporting systematic reviewsDeclaración PRISMA 2020: una guía actualizada para la publicación de revisiones sistemáticas]. Rev Panam Salud Publica. 2022;46:e112. 10.26633/RPSP.2022.112 PMC9798848 36601438

[15] Sterne JAC , Savović J, Page MJ, et al RoB 2: a revised tool for assessing risk of bias in randomised trials. BMJ. 2019;366:l4898. 10.1136/bmj.l4898 31462531

[16] Sterne JA , Hernán MA, Reeves BC, et al ROBINS-I: a tool for assessing risk of bias in non-randomised studies of interventions. BMJ. 2016;355:i4919. 10.1136/bmj.i4919 PMC5062054 27733354

[17] Bouhnik Y , Carbonnel F, Laharie D, et al; GETAID CREOLE Study Group. Efficacy of adalimumab in patients with Crohn’s disease and symptomatic small bowel stricture: a multicentre, prospective, observational cohort (CREOLE) study. Gut. 2018;67:53-60. 10.1136/gutjnl-2016-312581 PMC5754855 28119352

[18] Schulberg JD , Wright EK, Holt BA, et al Intensive drug therapy versus standard drug therapy for symptomatic intestinal Crohn’s disease strictures (STRIDENT): an open-label, single-centre, randomised controlled trial. Lancet Gastroenterol Hepatol. 2022;7:318-331. 10.1016/S2468-1253(21)00393-9 34890567

[19] Lovett GC , Schulberg JD, Hamilton AL, et al Crohn’s disease stricture response to treatment assessed with magnetic resonance imaging and intestinal ultrasound: STRIDENT randomized trial. Inflamm Bowel Dis. 2025;31:2777-2786. 10.1093/ibd/izaf073 PMC12558584 40339148

[20] Takenaka K , Kawamoto A, Hibiya S, et al Stricture dynamics and prognosis during biologic therapy in ileal Crohn’s disease: a real-world cohort. Published online February 10, 2026. Inflamm Bowel Dis. 10.1093/ibd/izag0122026 41665667

[21] Narula N , Wong ECL, Dulai PS, et al Outcomes of passable and non-passable strictures in clinical trials of Crohn’s disease: a post-hoc analysis. J Crohns Colitis. 2021;15:1649-1657. 10.1093/ecco-jcc/jjab045 33693522

[22] Pallotta N , Barberani F, Hassan NA, et al Effect of infliximab on small bowel stenoses in patients with Crohn’s disease. World J Gastroenterol. 2008;14:1885-1890. 10.3748/wjg.14.1885 PMC2700409 18350627

[23] Matsumoto H , Hata J, Yo S, et al Serial changes in intestinal stenotic stiffness in patients with Crohn’s disease treated with biologics: a pilot study using ultrasound shear wave elastography. Turk J Gastroenterol. 2023;34:1006-1013. 10.5152/tjg.2023.22768 PMC10645284 37603302

[24] Colombel JF , Sandborn WJ, Reinisch W, et al; SONIC Study Group. Infliximab, azathioprine, or combination therapy for Crohn’s disease. N Engl J Med. 2010;362:1383-1395. 10.1056/NEJMoa0904492 20393175

[25] Feagan BG , Sandborn WJ, Gasink C, et al; UNITI–IM-UNITI Study Group. Ustekinumab as induction and maintenance therapy for Crohn’s disease. N Engl J Med. 2016;375:1946-1960. 10.1056/NEJMoa1602773 27959607

[26] Ye BD , Pesegova M, Alexeeva O, et al Efficacy and safety of biosimilar CT-P13 compared with originator infliximab in patients with active Crohn’s disease: an international, randomised, double-blind, phase 3 non-inferiority study. Lancet. 2019;393:1699-1707. 10.1016/S0140-6736(18)32196-2 30929895

[27] Hall B , Holleran G, Chin JL, et al A prospective 52 week mucosal healing assessment of small bowel Crohn’s disease as detected by capsule endoscopy. J Crohns Colitis. 2014;8:1601-1609. 10.1016/j.crohns.2014.09.005 25257546

[28] Louis E , Boverie J, Dewit O, et al; Belgian IBD Research Group. Treatment of small bowel subocclusive Crohn’s disease with infliximab: an open pilot study. Acta Gastroenterol Belg. 2007;70:15-19. 17619533

[29] Holtmann M , Wanitschke R, Helisch A, et al [Anti-TNF antibodies in the treatment of inflammatory intestinal stenoses in Crohn’s disease]. Z Gastroenterol. 2003;41:11-17. 10.1055/s-2003-36677 12541166

[30] Kucharzik T , Taylor S, Allocca M, et al ECCO-ESGAR-ESP-IBUS guideline on diagnostics and monitoring of patients with inflammatory bowel disease: part 1. J Crohns Colitis. 2025;19:jjaf106. 10.1093/ecco-jcc/jjaf106 40741688

[31] Yanai H , Feakins R, Allocca M, et al ECCO-ESGAR-ESP-IBUS guideline on diagnostics and monitoring of patients with inflammatory bowel disease: part 2. J Crohns Colitis. 2025;19:jjaf107. 10.1093/ecco-jcc/jjaf107 40742985

[32] Merola E , Mereu S, Dore MP, Scaglione M. Dual-energy and photon-counting computed tomography in Crohn’s disease: towards quantitative imaging biomarkers. A systematic review. *J Crohns Colitis.* 2026; 20: jjaf231.558. 10.1093/ecco-jcc/jjaf231.558

