# Supplementary material for: The missing evidence: Stricturing Crohn’s disease in clinical trials, a systematic review
Source: Inflamm Bowel Dis. 2026 May 14;32(10):1898–909. doi: 10.1093/ibd/izag079 (PMC13626237; doi:10.1093/ibd/izag079)
Supplement: Supplementary file 1 [file izag079_supplementary_data.zip › Supplementary Material 1.pdf]

Supplementary Material 1. Search queries for the systematic review

PubMed

(("Crohn Disease"[Mesh] OR crohn*[tiab]) OR ("Inflammatory Bowel Diseases"[Mesh] OR IBD[tiab])) AND ("Biological Products"[Mesh] OR "Biological Therapy"[Mesh] OR "Antibodies, Monoclonal"[Mesh] OR "Janus Kinase Inhibitors"[Mesh] OR "Sphingosine 1-Phosphate Receptors"[Mesh] OR biologic*[tiab] OR "biological therapy"[tiab] OR "small molecule*"[tiab] OR "small-molecule*"[tiab] OR infliximab[tiab] OR adalimumab[tiab] OR certolizumab[tiab] OR golimumab[tiab] OR vedolizumab[tiab] OR ustekinumab[tiab] OR risankizumab[tiab] OR mirikizumab[tiab] OR guselkumab[tiab] OR brazikumab[tiab] OR etrolizumab[tiab] OR natalizumab[tiab] OR tofacitinib[tiab] OR upadacitinib[tiab] OR filgotinib[tiab] OR ozanimod[tiab] OR etrasimod[tiab] OR remicade[tiab] OR humira[tiab] OR cimzia[tiab] OR simponi[tiab] OR entyvio[tiab] OR stelara[tiab] OR skyrizi[tiab] OR omvoh[tiab] OR rinvoq[tiab] OR xeljanz[tiab] OR zeposia[tiab] OR velsipity[tiab] OR ((integrin[tiab] OR alpha4beta7[tiab] OR a4b7[tiab] OR "IL-23"[tiab] OR "IL-12/23"[tiab] OR "IL-12"[tiab]) AND (inhibitor[tiab] OR antagonist[tiab] OR antibody[tiab])) OR "anti-integrin"[tiab] OR "monoclonal antibod*"[tiab]) AND ("Clinical Trial"[Publication Type] OR "Randomized Controlled Trial"[Publication Type] OR "Controlled Clinical Trial"[Publication Type] OR "Clinical Trial, Phase II"[Publication Type] OR "Clinical Trial, Phase III"[Publication Type] OR "Clinical Trial, Phase IV"[Publication Type] OR "Pragmatic Clinical Trial"[Publication Type] OR random*[tiab] OR trial[tiab] OR "open-label"[tiab] OR "open label"[tiab] OR "Prospective Studies"[Mesh] OR prospect*[tiab] OR longitudinal[tiab] OR (cohort[tiab] AND (prospect*[tiab] OR longitudinal[tiab])) OR ("follow-up"[tiab] AND (prospect*[tiab] OR longitudinal[tiab] OR cohort[tiab])) OR placebo[tiab] OR blinded[tiab] OR double-blind*[tiab] OR single-blind*[tiab]) NOT (animals[mh] NOT humans[mh])

6391 sources

Scopus

((TITLE-ABS-KEY(crohn*) OR (TITLE-ABS-KEY("inflammatory bowel disease" OR IBD) AND TITLE-ABS-KEY(crohn*))) AND ((TITLE-ABS-KEY(biologic* OR "biological therapy" OR "small molecule*" OR "small-molecule*" OR "antibodies, monoclonal" OR "monoclonal antibod*" OR "anti-tnf" OR infliximab OR adalimumab OR certolizumab OR golimumab OR vedolizumab OR ustekinumab OR risankizumab OR mirikizumab OR guselkumab OR brazikumab OR

etrolizumab OR natalizumab OR tofacitinib OR upadacitinib OR filgotinib OR ozanimod OR etrasimod OR remicade OR humira OR cimzia OR simponi OR entyvio OR stelara OR skyrizi OR omvoh OR rinvoq OR xeljanz OR zeposia OR velsipity OR "anti-integrin" OR "jak inhibitor*" OR "janus kinase" OR "s1p modulator*" OR "s1p receptor modulator*" OR "sphingosine 1 phosphate")) OR (TITLE-ABS-KEY(integrin OR alpha4beta7 OR a4b7 OR "il-23" OR "il-12/23" OR "il-12") AND TITLE-ABS-KEY(inhibitor OR antagonist OR antibody)))) AND (TITLE-ABS-KEY(random* OR trial OR "clinical trial" OR "open label" OR "open-label" OR placebo OR blinded OR "double blind*" OR "double-blind*" OR "single blind*" OR "single-blind*" OR prospect* OR longitudinal OR "phase II" OR "phase III" OR "phase IV")) AND (LIMIT-TO (EXACTKEYWORD, "Humans")) AND EXCLUDE (EXACTKEYWORD, "Animals")

7609 sources

Web of Science

((TS=(crohn*)) OR (TS=("inflammatory bowel disease" OR IBD) AND TS=(crohn*))) AND TS=(biologic* OR "biological therapy" OR "small molecule*" OR "small-molecule*" OR "antibodies, monoclonal" OR "monoclonal antibod*" OR "anti-tnf" OR infliximab OR adalimumab OR certolizumab OR golimumab OR vedolizumab OR ustekinumab OR risankizumab OR mirikizumab OR guselkumab OR brazikumab OR etrolizumab OR natalizumab OR tofacitinib OR upadacitinib OR filgotinib OR ozanimod OR etrasimod OR remicade OR humira OR cimzia OR simponi OR entyvio OR stelara OR skyrizi OR omvoh OR rinvoq OR xeljanz OR zeposia OR velsipity OR "anti-integrin" OR "jak inhibitor*" OR "janus kinase" OR "s1p modulator*" OR "s1p receptor modulator*" OR "sphingosine 1 phosphate" OR ((integrin OR alpha4beta7 OR a4b7 OR "il-23" OR "il-12/23" OR "il-12") AND (inhibitor OR antagonist OR antibody))) AND (TS=(random* OR trial OR "clinical trial" OR "open label" OR "open-label" OR placebo OR blinded OR "double blind*" OR "double-blind*" OR "single blind*" OR "single-blind*" OR prospect* OR longitudinal) OR (TS=(cohort) AND TS=(prospect* OR longitudinal)) OR (TS=("follow up" OR "follow-up") AND TS=(prospect* OR longitudinal OR cohort)))

8305 sources

TOTALE: 22305
