# Supplementary material for: The missing evidence: Stricturing Crohn’s disease in clinical trials, a systematic review
Source: Inflamm Bowel Dis. 2026 May 14;32(10):1898–909. doi: 10.1093/ibd/izag079 (PMC13626237; doi:10.1093/ibd/izag079)
Supplement: Supplementary file 1 [file izag079_supplementary_data.zip › Supplementary Table clean.docx]

| **Supplementary Table 1. Major trials on biologics/small molecules for Crohn’s disease: details about stricturing phenotype.** | | | |
| --- | --- | --- | --- |
| **Study** | **Drug** | **Inclusion of stricturing CD** | **Outcomes reporting for stricturing CD** |
| **Anti-TNF antibodies** | | | |
| ACCENT I | Infliximab | Yes | No |
| Carlsen 2025 | Adalimumab | Yes | No |
| CHARM | Adalimumab | Only if asymptomatic | No |
| CLASSIC I / II | Adalimumab | Only if asymptomatic | No |
| CREOLE | Adalimumab | Yes | Yes |
| Huguet 2026 | Biosimilar CT-P13 | Unclear | - |
| INESTIMABLE | Infliximab | Yes | No |
| LADI | Adalimumab | Yes | No |
| Nomaru 2025 | Biosimilar CT-P13 | Yes | No |
| Papista 2025 | Biosimilar GP-1111 | Yes | No |
| PRECISE1/2 | Certolizumab pegol | Only if asymptomatic | No |
| PROFILE | Infliximab, immunomodulator | Yes | No |
| SONIC | Infliximab, azathioprine | Only if asymptomatic | No* |
| STRIDENT | Adalimumab, thiopurine | Yes | Yes |
| Ye et al. | Biosimilar CT-P13 | Yes | No* |
| **Anti-IL-12/IL-23 antibodies** | | | |
| AURORA study | Ustekinumab | Yes | No |
| Del Rosario Garcia 2026 | Ustekinumab | Yes | No |
| Matsumoto 2023 | Ustekinumab, Infliximab | Yes | Yes |
| POWER | Ustekinumab | Unclear | - |
| REScUE Study | Ustekinumab | Yes | No |
| SEAVUE | Ustekinumab vs. Adalimumab | No | - |
| STARDUST | Ustekinumab | Yes | - |
| UNITI–IM-UNITI Study | Ustekinumab | Yes | No* |
| **Anti-IL-23p19 antibodies** | | | |
| ADVANCE/MOTIVATE | Risankizumab | Unclear | - |
| Alzaabi 2025 | Risankizumab | Yes | No |
| FORTIFY | Risankizumab | Unclear | - |
| DIVERSITI | Guselkumab | Only if asymptomatic | No |
| LUCENT-1 | Mirikizumab | Only if asymptomatic | No |
| LUCENT-2 | Mirikizumab | Only if asymptomatic | No |
| SEQUENCE | Risankizumab | Unclear | - |
| STAR | Risankizumab | Yes | No |
| **Anti-integrin antibodies** | | | |
| GEMINI2 | Vedolizumab | No | - |
| GEMINI3 | Vedolizumab | Yes | No |
| Gros 2025 | Vedolizumab | Yes | No |
| Louis 2025 | Vedolizumab, other biologics | Yes | No |
| LOVE-CD | Vedolizumab | Unclear | - |
| PRIVEDO | Vedolizumab | Yes | No |
| Takenaka 2026 | Vedolizumab vs. ustekinumab vs. anti-TNF | Yes | Yes |
| Ukashi 2025 | Vedolizumab | Yes | No |
| VERSIFY | Vedolizumab | Unclear | - |
| VISIBLE 2 | Vedolizumab | No | - |
| **Anti-chemokin antibodies** | | | |
| Kobayashi 2025 | E6011 | No | - |
| **Small molecules** | | | |
| CELEST | Upadacitinib | Yes | No |
| DIVERSITY | Filgotinib | Only if asymptomatic | No |
| FITZROY | Filgotinib | Only if asymptomatic | - |
| Lüke 2026 | Upadacitinib | Yes | No |
| SELECTION | Filgotinib |  |  |
| U-ACHIEVE | Upadacitinib | Unclear | - |
| U-EXCEL | Upadacitinib | Unclear | - |
| U-EXCEED | Upadacitinib | Unclear | - |
| U-ENDURE | Upadacitinib | Unclear | - |
| YELLOWSTONE | Ozanimod | Only if asymptomatic | - |

* Post-hoc analysis by Narula et al.

|  |  |  |  |  |
| --- | --- | --- | --- | --- |
