# Supplementary material for: The missing evidence: Stricturing Crohn’s disease in clinical trials, a systematic review
Source: Inflamm Bowel Dis. 2026 May 14;32(10):1898–909. doi: 10.1093/ibd/izag079 (PMC13626237; doi:10.1093/ibd/izag079)
Supplement: Supplementary file 1 [file izag079_supplementary_data.zip › Supplementary Material 2.pdf]

Supplementary Material 2. Criteria for literature search

**Inclusion Criteria**	**Exclusion Criteria**
**Patient features**	
Diagnosis of stricturing CD	Diagnosis of non-stricturing CD
	Diagnosis of ulcerative colitis
Treatments with biological or small-molecule therapy	Diagnosis of stricturing CD treated with other therapies than biologics or small molecules
Age > 18 years	Age < 18 years
**Article type**	
Prospective studies (including clinical trials)	Review articles (narrative or systematic)
	Editorials, commentaries, letters to the editor
	Conference abstracts
	Guidelines
	Case reports and small case series
	Retrospective studies
	Preclinical investigations (no human participants)
	Prospective studies on structuring CD, not focusing on disease response to therapy

CD: Crohn's disease

PICO DEFINITION:

P adult patients with structuring Crohn's disease

I biological or small-molecule therapy

C other drug or placebo (in trials)

O disease response (activity scores? Improvement of radiological stenosis?)
